# Translating Visual Short-Term Memory Binding Tasks to Clinical Practice: From Theory to Practice

**DOI:** 10.3389/fneur.2020.00458

**Published:** 2020-06-10

**Authors:** Ivanna M. Pavisic, Aida Suarez-Gonzalez, Yoni Pertzov

**Affiliations:** ^1^Dementia Research Centre, Department of Neurodegenerative Diseases, UCL Queen Square Institute of Neurology, UCL, London, United Kingdom; ^2^UK Dementia Research Institute at University College London, UCL, London, United Kingdom; ^3^Department of Psychology, The Hebrew University of Jerusalem, Jerusalem, Israel

**Keywords:** binding, differential diagnosis, preclinical marker, translational research, clinical practice

## Introduction

Alzheimer's disease (AD) is the most common form of neurodegenerative dementia, accounting for 2/3 of all dementia cases and currently recognized as a global public health challenge. In 2015, 46.8 million people were estimated to have dementia, and this number is expected to almost double every 20 years reaching 75 million in 2030 and 131.5 million in 2050 (1). Early detection may offer the best chance of therapeutic success amid these raising numbers.

It is now recognized that a preclinical period may precede the symptomatic phase up to 25 years (2). The development of suitable behavioral markers to detect and track this stage is important, before more expensive and invasive biomarkers are used. One important line of AD research in the last decade has provided evidence that the ability to bind object features together in short-term memory (STM) is affected in AD even at asymptomatic stages (3, 4). In cognition, binding is the function that supports the integration of multiple elements together (5–7).

Popular in clinical psychology is the Memory Binding Test (MBT), which assesses the binding of a category cue (e.g., flower) to a word target [e.g., tulip; see (8) for detail on test] (9–11). However, its verbal nature causes susceptibility to semantic interference and cognitive reserve [CR; the ability to find alternative ways of performing a task, bypassing any deficits (12, 13)]. Instead, visual short-term memory (VSTM) binding relies on the integration of visual features and is less susceptible to semantic and verbal strategies. The focus of this article will be on binding of visual information across short time scales. Yet, before we tackle this in more depth, it is relevant to define a series of terms.

In clinical practice, “prodromal” is usually the period immediately preceding the onset of dementia, when patients might meet criteria for mild cognitive impairment (MCI) (14) and “preclinical” generally refers to the stage preceding this, before the onset of the clinical phenotype. Here, the term “preclinical AD” will be restricted to asymptomatic familial Alzheimer's disease (FAD)—a rare autosomal dominantly inherited variant of Alzheimer's and clinically healthy individuals (at time of testing) who, over time, developed AD dementia. We will provide brief theoretical reasoning for assessing VSTM binding in AD and a summary of the research lines in the field. In the context of clinical practice, we will also reflect on its use for the differential diagnosis of AD and as a tool for preclinical AD.

## Theoretical Reasoning

The “feature integration theory of attention” (6) suggests that when attention is focused on an object, all its attributes (e.g., shape, color, motion, and texture) (15) are rapidly bound into a unified representation that is then used by higher-level cognitive processes (16, 17). The different attributes on objects have to be bound together also after the perceptual stage, even when maintained in STM. STM binding is a cognitive function known to support the integration of features necessary to maintain a coherent representation of an object in immediate memory. Two types of VSTM binding deficits have been reported in AD: relational and conjunctive binding.

Some conceptual differences are mentioned below:

° Conjunctive binding: is the integration of features within an object, ultimately forming a single representations of the item with multiple elements (e.g., color and shape) (18). It does not seem to depend on the hippocampus (19–21) and appears supported by a network involving the entorhinal and perirhinal cortex as well as occipital–parietal regions (22–24).
° Relational binding: is the association of an object identity's to other “independent” features such as its location, context, or source [see (25)]. Successful performance relies on the integrity of the hippocampus (3) and appears to engage a network in which it plays a fundamental role (19, 20).

## Memory Binding in Relation to the Detection of Preclinical AD

Parra et al. were the first to suggest conjunctive binding as a preclinical marker of AD (26, 27). They used a change-detection task in which individuals detected a change between two consecutive displays. While a shift in one feature (e.g., color) was easily detected, when only the binding between features was changed (green circle and blue square green square and blue circle), asymptomatic presenilin 1 (*PSEN1*) carriers carrying an E280A mutation (causing FAD) were significantly impaired. Such tasks showed greater sensitivity than standard neuropsychological tests, which revealed no differences in this asymptomatic group compared to healthy controls.

More recently, Liang and colleagues (3) assessed relational binding by asking individuals to remember the identity and location of several objects. After a delay of a few seconds, subjects were required to report which one of two objects was presented and move the selected object to its correct location on a touch screen (28, 29). Asymptomatic FAD carriers, who performed similarly to matched controls on identifying the correct object, and in localization precision, exhibited more binding errors in which the correct object was localized precisely near the location of one of the other objects from the memory array (30).

## Memory Binding in Relation to the Differential Diagnosis Of Sporadic Ad

Binding tasks have also proven useful in the differential diagnosis of sporadic AD. In 2009, Parra and colleagues showed that AD patients had deficits in conjunctive binding (5). These were later revealed specific to AD relative to other neurological conditions [i.e., major depression (4) and non-AD dementias (30, 31)].

## Implications of Differences Between Relational and Conjunctive Binding for Clinical Use

Arguably, if relational and conjunctive binding rely on different brain mechanisms and regions, they may have distinct sensitivity to different disease stages. This is a debated topic in the field, as some believe that atrophy in the entorhinal and perirhinal cortex (conjunctive) precedes hippocampal changes (relational) (32) in AD. Others sustain both types of binding decline at a preclinical phase (33). There is insufficient evidence to determine whether one or both may act as effective predictive clinical markers. Convincing evidence would require longitudinal studies that follow individuals at risk of sporadic or familial AD from an asymptomatic stage through MCI (33).

## Memory Binding And Aging

Conjunctive binding appears preserved across lifespan in healthy aging (34, 35), whereas relational binding seems to decline as the hippocampus degenerates with age regardless of risk for AD (36, 37). Importantly, the basis of this differential impact is not fully understood and susceptibility to reduction of attentional resources in working memory needs further exploration (38). Whether suitable preclinical tests for AD should not show effects of healthy aging (39) is still debated among the scientific community. Arguably the highest predictive power of such tools could also be reached when comparing individuals who will develop AD to *age-matched controls* who do not share the same risk factors (33).

A summary of the findings showing sensitivity to preclinical and symptomatic AD is presented in Table 1.

**Table 1:** Summary of binding studies suggesting utility for the detection of preclinical AD and differential diagnosis of AD.

**Study**	**Cohort**	**Findings**	
		**Conjunctive binding**	**Relational binding**
**Preclinical AD**			
Parra et al. (26)	E280A single *PSEN1* mutation carriers of FAD.	Binding between object features such as color and shape or color and color showed greater sensitivity at identifying asymptomatic carriers compared to other traditional neuropsychological tasks.	
Liang et al. (3)	Asymptomatic *PSEN1* and *APP* carriers of FAD.		Asymptomatic carriers showed specific impairment in object-location binding despite intact memory for object identity and location.
**Differential diagnosis of AD from**			
Parra et al. (34)	Controls	The same binding task as Parra et al. (26); binding between object features were selectively disrupted in AD patients.	
Parra et al. (4)	Major depression (MD) patients.	The only significant effect found was in STM for shape–color binding and this was due to AD patients performing poorly in this condition compared to MD patients	
Parra et al. (27)	Controls (sporadic and familial AD patients).	Both patient groups exhibited color–color STM (within dimension) binding deficits.	
Della Sala et al. (49)	Non-AD dementia patients (i.e., frontotemporal dementia, vascular dementia, Lewy body dementia, and dementia associated with Parkinson's disease).	Only AD patients showed STM binding deficits. This deficit was observed even when memory for single features was at a similar level across patient groups.	
Zokaei et al. (31)	Parkinson's disease (PD) patients.		AD patients and not PD patients showed increased misbinding. Memory deficits in PD patients were associated with making more random errors or guesses compared to the AD population.

*In each category, findings are presented chronologically. Other binding studies relating to medial temporal lobe (MTL) dysfunction (50) [including the hippocampus (51)] suggest certain features such as object-location binding may provide a sensitive cognitive marker for MTL dysfunction in a range of diseases including AD (8). FAD, Familial Alzheimer's disease; PSEN1, presenilin 1; APP, amyloid precursor protein; STM, short-term memory*.

## Translational Potential of VSTM Binding

The effort to build on basic scientific research and develop therapies, screening, and diagnostics for individuals is central in the field of dementia research. In 2015, the European Society for Translational Medicine stated its goal was to “*combine disciplines, resources, expertise, and techniques within benchside, bedside and community to promote enhancements in prevention, diagnosis, and therapies*” (40). Crucial to clinical practice is the transition from (a) the initial confirmation of association with the outcome of interest (e.g., VSTM binding impairment is associated with a diagnosis of AD) to (b) acquiring sensitivity to a treatment or an intervention (e.g., VSTM binding deficits decline in response to a therapy) and (c) showing a “meaningful” change in patient behavior (e.g., change in VSTM binding score results in a different treatment strategy) (41).

Below, we reflect on the translational potential of VSTM binding in a clinical setting (before any regulatory approval is attempted) and outline the pros and cons in this context.

### Pros

- Diagnostic potential for asymptomatic and MCI stages of AD (3, 10, 26, 27).
- Potential as a behavioral marker for preclinical AD outperforms traditional memory measures that lack the same sensitivity (3, 26).
- Sensitivity in predicting cognitive decline and conversion from aMCI to AD (11, 41).
- Such computerized tasks are non-invasive, easy to administer, inexpensive, and easily portable.
- Usually do not require verbal reports and thus are not language-constrained.
- Impervious to education and intercultural differences (27).
- Reduced susceptibility to subjective interpretation of results compared to other traditional neuropsychology tasks (9).
- Reduced susceptibility to practice or learning effects as the repeated presentation of semantically meaningless stimuli such as polygon–color or fractal–location combinations is quickly overwritten (42, 43).
- The use of shape and color or abstract figures limits the variability in the way information is rehearsed, organized, and encoded. Controlled learning minimizes the use of individualized strategies increasing the probability that retrieval is based on direct access to what was learned in the first place (9).

### Cons

- There is greater need for validation of test–retest reliability of such tasks for the purpose of detecting and monitoring AD-related populations. This should ideally involve different research groups with ethnically diverse populations.
- Although some conjunctive binding tasks have proven impervious to differences in cognitive reserve (26), further validation and additional exploration on relational binding in healthy aging is necessary.
- Longitudinal studies are needed to establish the suitability for the detection of preclinical AD (in non-genetic forms) (33). This will provide greater validation for its use in preclinical sporadic AD.
- At present, the detection of preclinical AD or differential diagnosis of MCI or AD relies on group differences. Future studies should focus on its use at an individual screening level and evaluate the best threshold for determining “impaired performance”.
- Large-scale testing of non-clinical populations are lacking, and this is necessary for acquiring appropriate norms.
- There is no current evidence for a relationship between task performances and prediction of treatment outcomes (i.e., are patients with high VSTM binding scores more likely to benefit from specific medications or interventions?)
- There is a need for validation of task performances against AD biomarkers like abnormal amyloid beta–positron emission tomography (Aβ-PET), or abnormal Aβ in cerebrospinal fluid (CSF), or abnormal tau-PET, or abnormal tau in CSF—before considering using these approaches on their own.

## Discussion

The recent Food and Drug Administration guidelines suggest that biomarkers alone will not be sufficient as surrogate outcomes to show effectiveness of treatment (44). There is increasing recognition that therapies should be associated with changes in clinically meaningful endpoints (whether cognitive or functional) (45). It is therefore paramount to identify cognitive behavioral measures that are sensitive to detecting early disease states and ideally converge with biological markers of AD pathology. These become particularly important for identifying individuals at risk, monitoring disease progression, and ascertaining treatment efficacy (9, 46). If such tests were developed to identify cognitive deficits resulting from the earliest identifiable brain pathology in AD, such as the deposition of Aβ or abnormal phosphorylated tau (47, 48), measures could then serve as both highly powerful cognitive markers and, in turn, clinically significant end points.

In our opinion, there is currently insufficient evidence to determine the translational potential of VSTM binding tasks to clinical settings. However, the development of novel tests that are cognitively challenging, minimize variability in learning strategies, decrease the subjectivity to interpretation, and exploit vulnerabilities caused by AD is needed (9). VSTM binding tasks are indeed headed in this direction.

This leaves a number of questions unanswered: What are the most important characteristics of tests administered in a clinical setting? Do these characteristics vary depending on purpose (e.g., diagnosis, dementia incidence, and prognosis)? Is it far too early for the use of VSTM binding tasks in clinical practice?

Considering the current evidence, we propose that the greatest translational potential of VSTM binding tasks might lie on the preclinical stages of AD. Nonetheless, large-population-based studies, longitudinal designs, and correlations to biomarkers are paramount to validate this further. Lastly and crucial to clinical practice, it is yet to be determined if such tests are actionable, i.e., whether their prognostic and predictive value gives grounds for improving patient management.

## Author Contributions

IP: conceived a first version of the manuscript. IP, AS-G, and YP: conception and interpretation of the work, final approval of the manuscript, and agreement to be accountable for all aspects of the work in ensuring that questions related to the accuracy or integrity of any part of the work are investigated and resolved.

## Conflict of Interest

The authors declare that the research was conducted in the absence of any commercial or financial relationships that could be construed as a potential conflict of interest. The handling editor CB declared a past co-authorship with one of the authors, AS-G.

## Abbreviations

STM: short-term memory
FAD: familial Alzheimer's disease.
